# Regulation of Genes Involved in Heterocyst Differentiation in the Cyanobacterium *Anabaena* sp. Strain PCC 7120 by a Group 2 Sigma Factor SigC

**DOI:** 10.3390/life5010587

**Published:** 2015-02-16

**Authors:** Shigeki Ehira, Shogo Miyazaki

**Affiliations:** 1 Department of Biological Sciences, Graduate School of Science and Engineering, Tokyo Metropolitan University, Hachioji, Tokyo 192-0397, Japan; E-Mail: shogo_miyazaki_0502@yahoo.co.jp; 2 Precursory Research for Embryonic Science and Technology, Japan Science and Technology Agency, Kawaguchi, Saitama 332-0012, Japan

**Keywords:** sigma factor, transcriptional regulation, heterocyst differentiation, *Anabaena*

## Abstract

The filamentous cyanobacterium *Anabaena* sp. strain PCC 7120 differentiates specialized cells for nitrogen fixation called heterocysts upon limitation of combined nitrogen in the medium. During heterocyst differentiation, expression of approximately 500 genes is upregulated with spatiotemporal regulation. In the present study, we investigated the functions of sigma factors of RNA polymerase in the regulation of heterocyst differentiation. The transcript levels of *sigC*, *sigE*, and *sigG* were increased during heterocyst differentiation, while expression of *sigJ* was downregulated. We carried out DNA microarray analysis to identify genes regulated by SigC, SigE, and SigG. It was indicated that SigC regulated the expression of genes involved in heterocyst differentiation and functions. Moreover, genes regulated by SigC partially overlapped with those regulated by SigE, and deficiency of SigC was likely to be compensated by SigE.

## 1. Introduction

Heterocysts are terminally differentiated cells of filamentous cyanobacteria that are specialized for the fixation of atmospheric nitrogen. Upon limitation of combined nitrogen in the medium, particular vegetative cells differentiate into heterocysts with a semi-regular spacing of 10–15 cells [1,2]. In the filamentous cyanobacterium *Anabaena* sp. strain PCC 7120 (hereafter referred to as *Anabaena* PCC 7120), about 500 genes are upregulated with spatiotemporal regulation during the process of heterocyst development [3,4,5,6]. Two genes, *ntcA* and *hetR*, are necessary for heterocyst differentiation, and their induction by nitrogen deprivation is mutually dependent. NtcA, a transcriptional regulator that globally controls the nitrogen response in cyanobacteria, is activated by binding of 2-oxoglutarate, which is the central signaling molecule reflecting the cellular nitrogen status [7,8]. NtcA activates transcription of the *nrrA* gene, whose product in turn activates *hetR* expression [9,10]. *hetR* is a master gene of heterocyst differentiation [11,12]. Mutation in *hetR* blocks early steps in differentiation, whereas ectopic expression of *hetR* induces the formation of heterocysts irrespective of cellular nitrogen status. HetR is a DNA-binding protein [13], and its target genes were extensively determined by genomic searches of the HetR-binding sequence and deep sequencing of HetR-bound DNA *in vivo* [14,15]. The *hetR* gene is also required for upregulation of *ntcA* after nitrogen deprivation, although the molecular basis of *hetR*-dependent regulation of *ntcA* remains unknown [16,17].

The sigma factor of RNA polymerase is responsible for promoter recognition and determines the specificity of transcriptional initiation [18]. Bacteria respond to environmental changes by expressing specific sigma factors to induce particular sets of genes. In *Anabaena* PCC 7120, there are 12 genes encoding sigma factors, three of which (*sigB* (all7615), *sigB3* (all7608), and *sigB4* (all7179)) are carried on plasmids, and the remaining nine genes (*sigA* (all5263), *sigB2* (alr3800), *sigC* (all1692), *sigD* (alr3810), *sigE* (alr4249), *sigF* (all3853), *sigG* (alr3280), *sigI* (all2193), and *sigJ* (alr0277)) are carried on the chromosome [19]. We adopted the sigma factor nomenclature revised by Yoshimura *et al.* [20] in this study. Northern blot analysis showed that the transcript levels of *sigB*, *sigC*, and *sigE* (previously *sigF*) are increased by nitrogen deprivation [21,22]. In addition, it was indicated that transcription from the promoters of *sigC*, *sigE*, and *sigG* is activated in differentiating heterocysts [23]. Although the genes of group 2 sigma factors, namely, *sigB*, *sigB2* (previously *sigE*), *sigC*, *sigD*, and *sigE*, were inactivated, none of them were individually required for heterocyst differentiation [21,24]. However, heterocyst development in *sigB* and *sigC* mutants is delayed, and some double mutants of group 2 sigma factors take a longer time to establish diazotrophic growth. It is also indicated that the *sigE* gene is required for normal expression of some heterocyst-specific genes, such as *nifH*, *fdxH*, and *hglE2* [22]. Overexpression of *sigE* causes accelerated heterocyst development and an increased heterocyst frequency. These observations suggest that sigma factors are involved in the regulation of gene expression during heterocyst development.

In the present study, we identified genes regulated by SigC, SigE, and SigG of *Anabaena* PCC 7120, whose expression was increased after nitrogen deprivation in a *hetR*-dependent manner, using a DNA microarray. Inactivation of *sigC* resulted in downregulation of 58 genes at 8 h after nitrogen deprivation, including many genes involved in heterocyst differentiation, and retardation of heterocyst differentiation. The transcript levels of 68 genes were lower in the *sigE* disruptant than in the wild-type (WT) strain. It was indicated that many genes involved in heterocyst differentiation and function were regulated by both SigC and SigE. These results support the conclusion that SigC is involved in the expression of genes required for heterocyst differentiation, and that SigC and SigE have at least partially overlapping promoter specificities.

## 2. Materials and Methods

### 2.1. Bacterial Strains and Culture Conditions

*Anabaena* PCC 7120 and its derivatives were grown in BG-11 medium containing NaNO_3_ as a nitrogen source as described previously [6]. The mutant strains DRsigEK [25] and DRhetRS [26] were used as the *sigE* and *hetR* disruptants in this study. Liquid cultures were bubbled with air containing 1.0% (v/v) CO_2_. For nitrogen deprivation experiments, filaments grown in BG-11 medium until they reached an OD_750_ of 0.4–0.5 were washed twice with sterile water and then resuspended in nitrogen-free BG-11 medium (BG-11_0_). Spectinomycin and neomycin were added to the medium at a final concentration of 10 and 30 µg mL^−1^, respectively, when required.

### 2.2. Mutant Construction

All primers used in this study (Table 1) were designed based on genome data from CyanoBase (Fujisawa *et al.*, 2014). DNA fragments upstream and downstream of the *sigC* gene were amplified by PCR using the primer pair sigC-5F and sigC-5R, and the primer pair sigC-3F and sigC-3R (Table 1), respectively. The upstream fragment was cloned between the SacI and BamHI sites of pBluescript II KS^+^ (Agilent Technologies), and then the downstream fragment was cloned between the BamHI and XhoI sites. A spectinomycin resistance cassette excised by digestion with BamHI from the plasmid pDW9 [27] was inserted into the BamHI site between the upstream and downstream fragments. The SacI-XhoI fragment was excised from the resultant plasmid and cloned between the SacI and XhoI sites of pRL271 [28] to construct pRsigCS. The pRsigGS plasmid was constructed as described for pRsigCS using the primer pair sigG-5F and sigG-5R, and the primer pair sigG-3F and sigG-3R (Table 1). pRsigCS and pRsigGS were transferred into *Anabaena* PCC 7120 by conjugation according to the method of Elhai *et al.* [29] to construct the deletion mutants of *sigC*, DRsigCS and *sigG*, DRsigGS, respectively.

**Table 1:** Primers used in this study.

Primer	Sequence (5'- 3')
sigC-5F	TAGAGCTCGCGGACTCACAGAAATGGTT
sigC-5R	TAGGATCCAATGGCGATATCAGGGTCT
sigC-3F	TAGGATCCTCGCAACCTTATCCGTGACT
sigC-3R	ATCTCGAGTTTGGCAGTCCAGTAGGTGA
sigG-5F	AGGAGCTCACGTCCATGATCAAACCAA
sigG-5R	TAGGATCCGTGGTTCGAGAGTTTGTCA
sigG-3F	ACGGATCCTTGCCGAAATCACAGGTGTA
sigG-3R	ATCTCGAGGGCGTGGGTATATTTGATG
RTrrn16SF2	GCAAGTCGAACGGTCTCTTC
RTrrn16SR2	GGTATTAGCCACCGTTTCCA
RTsigA-F	TTGTTGCTCGCTGATGATGG
RTsigA-R	TTCTTGCTTTGTGTCCGACG
RTsigB2-F	ACACCCACACAGAAGACACA
RTsigB2-R	TCTTTAGCGTCAATCAGCGAC
RTsigC-F	ACCTGGAGCCATAGAGACGA
RTsigC-R	CATCCACCGACAAATCACTG
RTsigD-F	AGCGTAGAAGAGTGGGCAAA
RTsigD-R	GGATACCACTAGCCGCAAGT
RTsigE-F	TGGCACGTTATCCACTGCTA
RTsigE-R	GTCGGATGTTGCCCTATTTG
RTsigF-F	TTGCGGGAACAATACAACCG
RTsigF-R	CCATCTTGCACGGGTACATC
RTsigG-F	TTGATGCGAGGTGTCCAGAA
RTsigG-R	GGTTTGATAGCGGCGCAATA
RTsigI-F	GATTCGGCGGCATTAAGTGT
RTsigI-R	GCTTCTTGGGAATCAGCCAG
RTsigJ-F	GGCAGCAAGTGAGTCCTCTA
RTsigJ-R	GCCGGTGTGTAATTGAACCA
RTfbp-F	CAACCTTATCCCGTCACGTC
RTfbp-R	GCGACGGGCAACTAATTTAC
RTtalB-F	GACCACCAATCCCTCTCTGA
RTtalB-R	AAGCGAGGGAGACAATTTGA
RTcoxB3-F	AAGGGCCGACAGCATTAGTA
RTcoxB3-R	ATACCCACGCCCATTGTTTA
RTcoxA2-F	CAACGCATTCATGACCAATC
RTcoxA2-R	AAGGTGGGTAAGCAGTCCAA
RThepA-F	CAGGAATTAGCTGGGTTGACA
RThepA-R	ATTGAAGGTAGCACGCATCC
RThepB-F	AAATTTATCGCGCCAACAAG
RThepB-R	CTCCGACACGATGCACTAAA
RTntcA-F	CAAGATAAGGCCCTAGCAAATG
RTntcA-R	TCCGACTTGTTTCCTGTCAAC
RT4160-F	TCATGACTAGCCAACCCACA
RT4160-R	TACTGCTTCCAGCACGCTTA

### 2.3. DNA Microarray Analysis

Cells used for RNA extraction were collected by centrifugation at 4 °C. Before centrifugation, the culture medium was rapidly chilled by adding crushed ice to eliminate additional effects during centrifugation. Collected cells were immediately frozen in liquid nitrogen and stored at –80 °C until use. Total RNA was extracted from whole filaments as described by Pinto *et al.* [30] and treated with DNase I (Takara Bio, Shiga, Japan). Global gene expression was analyzed using the *Anabaena* oligonucleotide microarray as described previously [6]. Microarray analyses were carried out with three sets of RNA samples (DRsigCS and DRsigEK) or two sets of RNA samples (DRsigGS) isolated from independently grown cultures. Two hybridization reactions were performed with different combinations of Cy-dyes for each set of RNA samples. Genes whose transcript levels significantly differed (*p* < 0.05; Student’s *t*-test) by at least 2-fold were determined. The microarray data were deposited in the KEGG Expression Database (accession numbers ex0001955 to ex0001970).

### 2.4. Quantitative Reverse Transcription PCR (qRT-PCR)

cDNA was synthesized from 1 µg of total RNA with random hexamer primers using a PrimeScript 1st strand cDNA Synthesis Kit (Takara Bio, Shiga, Japan). qRT-PCR was performed with a Thermal Cycler Dice Real-time System (TP900; Takara Bio, Shiga, Japan) in a 20 µL reaction mixture containing 10 µL of THUNDERBIRD SYBR qPCR Mix (Toyobo, Osaka, Japan), 0.2 µM of each gene-specific forward and reverse primer (Table 1), and cDNA. Relative ratios were normalized against the value for 16S rRNA and are represented as means of triplicate experiments.

### 2.5. Acetylene Reduction Assays

The acetylene reduction assays were performed as described previously [25]. The concentration of ethylene was measured by GC-2014 gas chromatography (Shimadzu, Kyoto, Japan). The chlorophyll *a* content of cultures was determined by the method of Mackinney [31], and the acetylene reduction rates were normalized to the chlorophyll *a* content.

## 3. Results and Discussion

### 3.1. Nitrogen-Regulated Expression of Genes Encoding Sigma Factors

**Figure 1:**
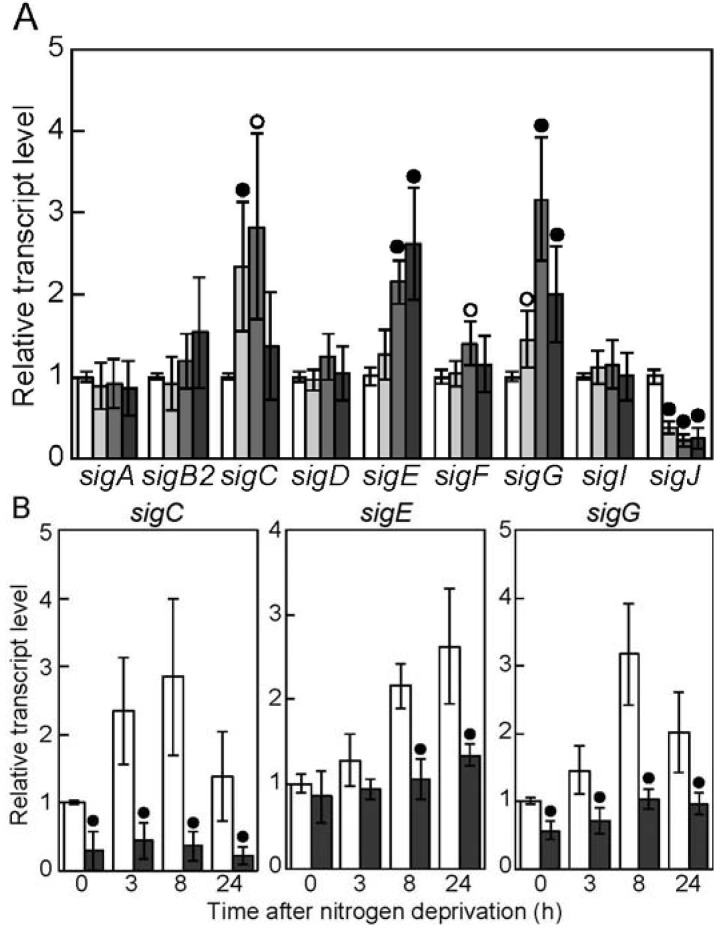
Changes in the transcript levels of genes encoding sigma factors after nitrogen deprivation. (**A**) The relative transcript levels of sigma factor genes before (white bars) and 3 h (light gray bars), 8 h (gray bars), and 24 h (dark gray bars) after nitrogen deprivation were determined by quantitative reverse transcription PCR. The transcript levels at 0 h were set to 1 for each gene. The *t*-test was used to compare 0 h and each time point, and data that represent a significant difference (*p* < 0.01 or 0.05) are marked with black or white circles; (**B**) the transcript levels of *sigC*, *sigE*, and *sigG* were determined in the wild-type (WT) strain (white bars) and the *hetR* disruptant (gray bars). The transcript levels at 0 h in the WT strain were set to 1. The *t*-test was used to compare the WT strain and the *hetR* disruptant, and data that represent a significant difference (*p* < 0.01) are marked with black circles.

Changes in the expression of sigma factor genes on the chromosome of *Anabaena* PCC 7120 during heterocyst differentiation were determined by qRT-PCR (Figure 1A). The transcript levels of *sigC*, *sigE*, and *sigG* increased during heterocyst differentiation, while *sigJ* was downregulated. Expression of *sigA*, *sigB2*, *sigD*, *sigF*, and *sigI* did not respond to nitrogen deprivation. The *sigC* transcript level was increased within 3 h after nitrogen deprivation and remained high until 24 h. The *sigE* and *sigG* transcript levels were increased at 8 h after nitrogen deprivation. These results are consistent with observations using GFP reporter strains, in which the *gfp* gene on the multi-copy plasmid was expressed from each promoter of sigma factor genes [23], although the transcript level of *sigE* increased earlier than the fluorescence level of GFP expressed from the *sigE* promoter. The *sigC*, *sigE*, and *sigG* genes are specifically upregulated in differentiating heterocysts [23], and the *sigE* transcript level is higher in mature heterocysts than in vegetative cells [32]. To determine the correlation between expression of *sigC*, *sigE*, and *sigG* and heterocyst differentiation, expression of these genes was determined in the *hetR* disruptant DRhetRS (Figure 1B). Upregulation of these genes by nitrogen deprivation was abolished in DRhetRS, indicating that expression of *sigC*, *sigE*, and *sigG* is developmentally regulated and that *hetR* is necessary for their upregulation. In the unicellular cyanobacterium *Synechocystis* sp. PCC 6803, *sigE* is also induced by nitrogen starvation under the control of NtcA, while no induction of *sigC* and *sigG* is observed [33,34]. In *Anabaena* PCC 7120, NtcA and NrrA, which are highly expressed in differentiating heterocysts [6,35], bind to the promoter regions of *sigE* and are involved in the regulation of *sigE* expression [22,25]. In response to nitrogen deprivation, *hetR* is necessary for upregulation of *ntcA*, and NtcA induces the expression of *nrrA* [9,17]. The *hetR* gene is likely to affect the expression of *sigE* via NtcA and NrrA, although it remains to be revealed how *hetR* regulates expression of *ntcA*. A genomic survey of the HetR-binding sequence and deep sequencing of HetR-bound DNA in vivo did not show an interaction between HetR and the promoters of *sigC* and *sigG* [14,15]. Thus, the mechanisms by which *hetR* regulates *sigC* and *sigG* remain unknown. We constructed mutants of group 3 (*sigF* and *sigJ*) and ECF (*sigG* and *sigI*) sigma factors; however, all of these were able to form heterocysts and grow diazotrophically (unpublished data). Combined with the results of inactivation of group 2 sigma factors [21,24], no group 2, group 3, or ECF sigma factor on the chromosome of *Anabaena* PCC 7120 is individually required for heterocyst differentiation.

### 3.2. The sigC Gene Is Required for Normal Induction of Genes Involved in Heterocyst Differentiation

DNA microarray analysis was carried out to identify genes regulated by SigC and SigG using the *sigC* disruptant DRsigCS and the *sigG* disruptant DRsigGS. Gene expression during heterocyst differentiation drastically changed after 8 h of nitrogen deprivation [5]; therefore, gene expression patterns of the WT strain and DRsigCS or DRsigGS after 8 h of nitrogen deprivation were compared.

**Figure 2:**
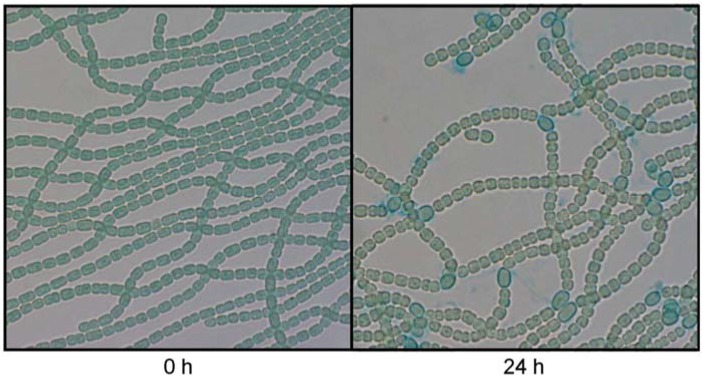
Heterocyst development after nitrogen deprivation in the *sigC* disruptant. Micrographs were taken before and 24 h after nitrogen deprivation. The polysaccharide layer of heterocysts was stained with Alcian blue.

The transcript levels of 58 genes were lower in DRsigCS than in the WT strain (Table 2). Downregulated genes included genes encoding enzymes of the oxidative pentose phosphate (OPP) pathway (*talB*, *talA*, and *fbp*), those encoding terminal respiratory oxidases (*coxB2*, *coxA2*, and cox*B3*), and those involved in the synthesis of heterocyst envelope polysaccharide (HEP). Moreover, the transcript level of the *ntcA* gene was decreased by *sigC* disruption. The OPP pathway is required for nitrogen fixation in heterocysts [36], and genes involved in the OPP pathway are upregulated in heterocysts [32]. The *coxBAC2* operon encodes an *aa_3_*-type cytochrome *c* oxidase, and the *coxBAC3* operon encodes the alternative respiratory terminal oxidase [37]. Both operons are specifically expressed in heterocysts, and either oxidase is necessary for aerobic nitrogen fixation [37,38]. Synthesis of HEP depends on a cluster of genes, the HEP island (alr2825 to alr2841), and some genes, such as *hepB* and all4160, that are distant from the HEP island in the genome [5,13,39]. All genes in the HEP island (the relative ratio of the alr2840 transcript level in DRsigCS to that in the WT strain was −0.96 with a *p* value of 0.0015), *hepB* (the relative ratio was −0.96 with a *p* value of 0.0034), and all4160 were downregulated by *sigC* disruption (Table 2). It was indicated that SigC regulates genes involved in heterocyst differentiation and functions, which are highly expressed in heterocysts. In DRsigCS, heterocysts were observed after 24 h of nitrogen deprivation (Figure 2). However, the nitrogenase activity in DRsigCS at 24 h was reduced to about 30% of that in the WT strain (Table 3). The nitrogenase activity in DRsigCS increased to the level in the WT strain after 48 h of nitrogen deprivation (Table 3), indicating that *sigC* disruption did not block heterocyst differentiation, but delayed heterocyst development as previously reported [21,24].

**Table 2:** Genes downregulated by *sigC* disruption.

ORF No.	Gene	Product	Δ*sigC*/WT ^a^	*p* ^b^
all0406	*-*	Unknown protein	−1.16	3.6 × 10^−2^
all0438	*-*	Serine/threonine kinase with WD-40 repeat	−1.53	5.1 × 10^−3^
asl0597	*-*	Hypothetical protein	−1.01	1.4 × 10^−2^
all0918	*-*	Unknown protein	−1.19	4.5 × 10^−2^
all0919	*-*	Probable glycosyltransferase	−1.96	5.9 × 10^−4^
all1101	*-*	Ferrichrome iron receptor	−1.01	3.0 × 10^−2^
alr1112	*-*	Probable transglycosylase	−1.04	3.4 × 10^−2^
alr1276	*-*	Putative acetyl transferase	−1.14	2.7 × 10^−3^
asr1405	*-*	Hypothetical protein	−1.11	6.3 × 10^−3^
asr1408	*nifZ*	Iron-sulfur cofactor synthesis protein	−1.29	2.9 × 10^−3^
all1424	*-*	Unknown protein	−1.19	9.5 × 10^−5^
asl1778	*-*	Unknown protein	−1.54	2.9 × 10^−5^
alr2323	*htpG*	Heat shock protein	−1.04	4.3 × 10^−2^
alr2405	*isiB*	Flavodoxin	−1.50	8.7 × 10^−3^
alr2514	*coxB2*	Cytochrome c oxidase subunit II	−1.37	2.2 × 10^−2^
alr2515	*coxA2*	Cytochrome c oxidase subunit I	−1.62	1.4 × 10^−3^
asr2523	*-*	Unknown protein	−1.24	2.4 × 10^−3^
all2563	*talB*	Transaldolase	−1.13	1.9 × 10^−2^
alr2582	*-*	Hypothetical protein	−1.41	9.5 × 10^−3^
all2637	*-*	Unknown protein	−1.08	3.5 × 10^−2^
all2655	*-*	Unknown protein	−1.16	1.2 × 10^−2^
alr2730	*-*	Hypothetical protein	−1.12	1.3 × 10^−2^
alr2731	*coxB3*	Cytochrome c oxidase subunit II	−1.11	2.0 × 10^−3^
alr2818	*hetP*	Heterocyst differentiation protein	−1.39	1.7 × 10^−3^
alr2822	*-*	Hypothetical protein	−1.48	3.9 × 10^−4^
alr2823	*-*	Hypothetical protein	−1.58	1.3 × 10^−4^
alr2824	*-*	Hypothetical protein	−2.24	4.9 × 10^−4^
alr2825	*-*	Glucose-1-P cytidylyltransferase	−1.38	2.1 × 10^−4^
alr2826	*-*	Hypothetical protein	−1.77	3.8 × 10^−4^
alr2827	*-*	Putative epimerase/dehydratase	−1.32	4.2 × 10^−4^
alr2828	*-*	Unknown protein	−1.92	7.3 × 10^−4^
alr2829	*-*	Unknown protein	−1.70	3.1 × 10^−4^
alr2830	*rfbC*	dTDP-4-dehydrorhamnose 3,5-epimerase	−1.40	3.1 × 10^−4^
alr2831	*-*	Probable NAD(P)-dependent oxidoreductase	−1.54	1.5 × 10^−3^
alr2832	*-*	Putative glycosyltransferase	−1.16	9.6 × 10^−5^
alr2833	*-*	Hypothetical protein	−1.44	3.7 × 10^−3^
alr2834	*hepC*	Similar to glycosyltransferase	−1.65	2.7 × 10^−5^
alr2835	*hepA*	ATP-binding protein of ABC transporter	−1.54	5.1 × 10^−3^
alr2836	*-*	Glycosyltransferase	−1.38	3.9 × 10^−5^
alr2837	*-*	Glycosyltransferase	−1.07	3.2 × 10^−3^
alr2838	*-*	Unknown protein	−1.00	2.7 × 10^−2^
alr2839	*-*	Glycosyltransferase	−1.13	5.6 × 10^−3^
alr2841	*-*	Unknown protein	−1.21	1.9 × 10^−3^
alr2887	*-*	Hypothetical protein	−1.00	2.0 × 10^−2^
all3420	*-*	Carboxyl-terminal processing protease	−2.65	1.5 × 10^−3^
all3780	*-*	Similar to kinesin light chain	−1.08	3.5 × 10^−2^
all3999	*-*	Unknown protein	−1.37	9.7 × 10^−3^
all4000	*-*	Photosystem II CP43 protein PsbC homolog	−1.57	1.8 × 10^−2^
all4001	*isiA*	Photosystem II chlorophyll a-binding protein	−2.59	1.4 × 10^−2^
all4002	*-*	Photosystem II CP43 protein PsbC homolog	−2.18	1.3 × 10^−2^
all4003	*-*	Photosystem II CP43 protein PsbC homolog	−1.87	1.1 × 10^−2^
all4020	*talA*	Transaldolase	−1.10	2.4 × 10^−2^
all4021	*fbp*	Fructose 1,6-bisphosphatase	−1.35	9.2 × 10^−3^
all4160	*-*	Probable glycosyltransferase	−1.12	1.4 × 10^−3^
asl4206	*rps17*	30S ribosomal protein S17	−1.10	4.8 × 10^−2^
alr4392	*ntcA*	Nitrogen-responsive global transcriptional regulator	−1.24	6.4 × 10^−3^
alr4984	*-*	Unknown protein	−1.39	1.3 × 10^−4^
alr5256	*-*	Biotin acetyl-CoA carboxylase ligase	−1.05	5.4 × 10^−4^

^a^ Relative ratios of transcript levels in the *sigC* disruptant to those in the wild-type strain at 8 h after nitrogen deprivation are shown in the base 2 logarithm; ^b^ *p* values for the relative ratios were determined by the Student’s *t-*test.

**Table 3:** Nitrogenase activities of the wild-type (WT) strain, the *sigC* disruptant, and the *sigE* disruptant.

Strain	Nitrogenase activity ^a^	
	(µmol C_2_H_4_/mg chl*a*/h)	
	24 h	48 h
WT	19.4 ± 7.0	17.4 ± 2.5
DRsigCS	6.0 ± 3.0	19.0 ± 5.5
DRsigEK	19.3 ± 6.3	11.0 ± 2.4

^a^ Nitrogenase activities were determined at 24 h and 48 h after nitrogen deprivation. Means ± S.D. of at least four independent experiments are shown.

**Figure 3:**
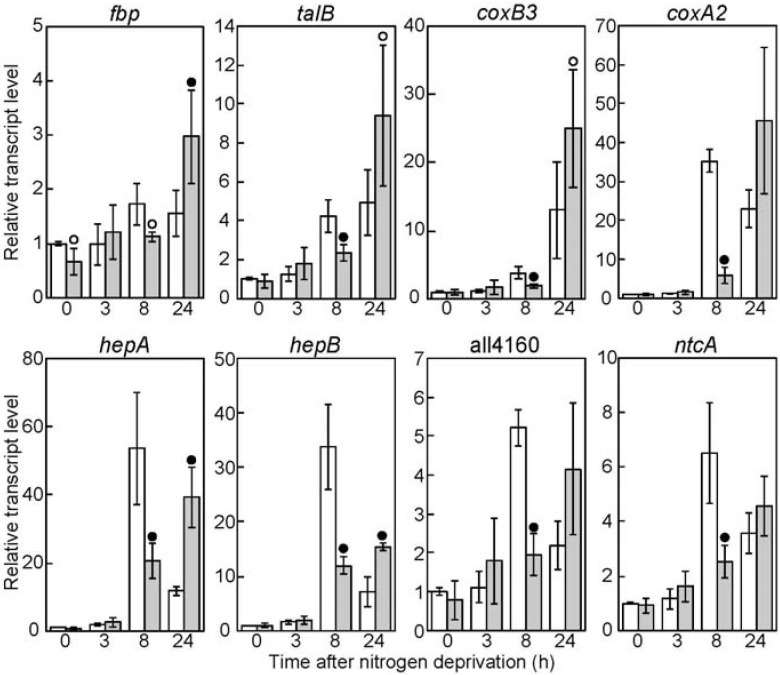
Changes in the transcript levels of genes downregulated by *sigC* disruption during heterocyst differentiation. The transcript levels of *fbp*, *talB*, *coxB3*, *coxA2*, *hepA*, *hepB*, all4160, and *ntcA* were determined in the wild-type (WT) strain (white bars) and the *sigC* disruptant (gray bars). The transcript levels at 0 h in the WT strain were set to 1. The *t*-test was used to compare the WT strain and the *sigC* disruptant, and data that represent a significant difference (*p* < 0.01 or 0.05) are marked with black or white circles.

We further analyzed the effects of *sigC* inactivation on gene expression during a time series of 24 h of heterocyst differentiation by qRT-PCR. The transcript levels of *fbp*, *talB*, *coxB3*, *coxA2*, *hepA*, *hepB*, all4160, and *ntcA* in DRsigCS after 8 h of nitrogen deprivation were lower than those in the WT strain (Figure 3). In the WT strain, expression of all these genes, except for that of *coxB3*, was constant or decreased between 8 h and 24 h, while their expression in DRsigCS was increased, which could be due to transcription by RNA polymerase with a sigma factor other than SigC. It is indicated that SigC is required for upregulation of genes expressed in heterocysts, although another sigma factor(s) can compensate for the deficiency of SigC.

The transcript levels of 14 genes were lower in DRsigGS than in the WT strain after 8 h of nitrogen deprivation (Table 4). No genes known to be involved in heterocyst differentiation were downregulated by *sigG* disruption. Further detailed analyses are required to clarify the relationship between the *sigG* gene and heterocyst differentiation.

**Table 4:** Genes downregulated by *sigG* disruption.

ORF No.	Gene	Product	Δ*sigG*/WT ^a^	*p* ^b^
alr2313	*-*	Unknown protein	−2.56	4.7 × 10^−5^
all2564	*pyk1*	Pyruvate kinase	−1.74	1.3 × 10^−2^
alr3281	*-*	Hypothetical protein	−1.05	7.3 × 10^−4^
alr3301	*-*	Unknown protein	−1.34	5.6 × 10^−3^
alr3445	*-*	Hypothetical protein	−1.24	9.4 × 10^−4^
alr3608	*-*	Similar to endoglucanase	−1.12	3.0 × 10^−3^
alr3816	*-*	Unknown protein	−1.41	3.6 × 10^−3^
alr3817	*-*	Unknown protein	−1.09	6.6 × 10^−3^
all3983	*-*	Similar to surface layer protein	−1.79	5.7 × 10^−3^
all4254	*-*	Unknown protein	−1.07	1.6 × 10^−4^
all4427	*-*	Similar to phytanoyl-CoA hydroxylase	−1.03	6.5 × 10^−4^
all4523	*-*	Hypothetical protein	−1.32	2.3 × 10^−3^
all4830	*-*	Mannosyl transferase	−1.76	9.4 × 10^−4^
alr5340	*-*	Hypothetical protein	−1.38	4.1 × 10^−3^

^a^ Relative ratios of transcript levels in the *sigG* disruptant to those in the wild-type strain at 8 h after nitrogen deprivation are shown in the base 2 logarithm; ^b^ *p* values for the relative ratios were determined by the Student’s *t-*test.

### 3.3. Genes Regulated by SigC Partially Overlap with Those Regulated by SigE

It was indicated that *sigE* is involved in the regulation of some late-stage heterocyst-specific genes, such as *nifH* and *fdxH*, which are expressed between 18 h and 24 h [22]. However, the transcript level of *sigE* was increased within 8 h after nitrogen deprivation (Figure 1). To determine the role of *sigE* at the earlier stage of heterocyst differentiation, gene expression patterns in the WT strain and the *sigE* disruptant DRsigEK after 8 h of nitrogen deprivation were compared.

The transcript levels of 68 genes were lower in DRsigEK than in the WT strain, including the *xfp* operon whose expression is activated by *sigE* [25] (Table 5). In addition to *nif* genes (*nifV1*, *nifZ*, and *nifU*), genes required for the synthesis of the glycolipid layer of the heterocyst envelop, namely, *devBC* [40] and *hglT* [41], and the *hetN* gene, which suppresses heterocyst differentiation [42], were downregulated by disruption of *sigE*. These genes were not identified as being downregulated in DRsigCS, while *coxBAC2*, *talB*, and genes involved in the synthesis of HEP were downregulated in DRsigEK as well as in DRsigCS. It is indicated that *sigE* is also involved in the regulation of genes that are induced within 8 h after nitrogen deprivation and that genes regulated by SigC and SigE partially overlap. To ascertain the role of *sigE* in heterocyst development, nitrogenase activities were determined. Nitrogenase activity in DRsigEK after 24 h of nitrogen deprivation was comparable with that in the WT strain, while nitrogenase activity in DRsigEK was reduced to 60% of that in the WT strain after 48 h (Table 3). Thus, single disruption of *sigE* had little effect on heterocyst development. However, a previous study found that heterocyst formation takes a longer time in the double mutant of *sigC* and *sigE* than in the single mutants of *sigC* or *sigE* [24]. These results support the idea that SigC and SigE have at least partially overlapping promoter specificities.

**Table 5:** Genes downregulated by *sigE* disruption.

ORF No.	Gene	Product	Δ*sigE*/WT ^a^	*p* ^b^
asl0046	*-*	Hypothetical protein	−1.59	1.2 × 10^−3^
all0178	*-*	Flavoprotein	−1.00	1.5 × 10^−3^
all0349	*-*	Unknown protein	−1.24	2.0 × 10^−3^
all0438	*-*	Serine/threonine kinase with WD-40 repeat	−1.24	6.7 × 10^−3^
all0916	*-*	ABC transporter ATP-binding subunit	−1.05	4.0 × 10^−2^
all0917	*-*	ABC transporter permease protein	−1.22	6.6 × 10^−4^
all0918	*-*	Unknown protein	−1.12	6.2 × 10^−4^
all0919	*-*	Probable glycosyltransferase	−1.21	2.5 × 10^−5^
alr1407	*nifV1*	Homocitrate synthase	−1.41	2.2 × 10^−4^
asr1408	*nifZ*	Iron-sulfur cofactor synthesis protein	−1.06	5.3 × 10^−3^
all1425	*-*	Unknown protein	−1.07	4.9 × 10^−2^
all1456	*nifU*	Nitrogen fixation protein	−1.00	4.5 × 10^−2^
all1814	*-*	Unknown protein	−1.03	1.3 × 10^−2^
alr2514	*coxB2*	Cytochrome c oxidase subunit II	−1.40	4.4 × 10^−2^
alr2515	*coxA2*	Cytochrome c oxidase subunit I	−1.62	2.8 × 10^−2^
alr2516	*coxC2*	Cytochrome c oxidase subunit III	−1.59	2.4 × 10^−2^
alr2517	*-*	Hypothetical protein	−1.13	7.1 × 10^−3^
asr2523	*-*	Unknown protein	−1.36	1.8 × 10^−4^
alr2524	*-*	Unknown protein	−2.04	2.5 × 10^−3^
all2563	*talB*	Transaldolase	−1.23	1.1 × 10^−4^
all2564	*pyk1*	Pyruvate kinase	−1.45	2.7 × 10^−3^
all2566	*gap1*	Glyceraldehyde-3-phosphate dehydrogenase	−1.23	6.8 × 10^−6^
all2567	*xfp*	Phosphoketolase	−1.33	7.3 × 10^−5^
all2571	*-*	Unknown protein	−1.37	6.8 × 10^−4^
alr2582	*-*	Hypothetical protein	−1.73	1.3 × 10^−3^
all2635	*-*	Polyketide synthase type I	−1.19	4.0 × 10^−2^
all2647	*-*	Microcystin synthetase B	−1.16	2.6 × 10^−2^
all2650	*-*	ABC transporter ATP-binding protein	−1.39	3.4 × 10^−2^
all2652	*-*	Hypothetical protein	−1.03	2.8 × 10^−2^
all2655	*-*	Unknown protein	−1.45	3.7 × 10^−2^
alr2729	*-*	Hypothetical protein	−1.33	3.5 × 10^−4^
alr2730	*-*	Hypothetical protein	−1.12	4.5 × 10^−5^
alr2818	*hetP*	Heterocyst differentiation protein	−1.34	1.2 × 10^−4^
alr2822	*-*	Hypothetical protein	−1.42	3.6 × 10^−3^
alr2823	*-*	Hypothetical protein	−1.60	3.6 × 10^−3^
alr2824	*-*	Hypothetical protein	−1.93	1.2 × 10^−4^
alr2825	*-*	Glucose-1-P cytidylyltransferase	−1.49	3.0 × 10^−3^
alr2826	*-*	Hypothetical protein	−1.75	4.7 × 10^−6^
alr2827	*-*	Putative epimerase/dehydratase	−1.42	1.0 × 10^−4^
alr2828	*-*	Unknown protein	−1.87	4.4 × 10^−5^
alr2829	*-*	Unknown protein	−1.92	3.1 × 10^−5^
alr2830	*rfbC*	dTDP-4-dehydrorhamnose 3,5-epimerase	−1.69	3.9 × 10^−5^
alr2831	*-*	Probable NAD(P)-dependent oxidoreductase	−1.19	4.0 × 10^−2^
alr2832	*-*	Putative glycosyltransferase	−1.69	1.0 × 10^−6^
alr2833	*-*	Hypothetical protein	−1.61	1.2 × 10^−5^
alr2834	*hepC*	Similar to glycosyltransferase	−1.94	1.3 × 10^−4^
alr2835	*hepA*	ATP-binding protein of ABC transporter	−1.74	1.4 × 10^−5^
alr2836	*-*	Glycosyltransferase	−1.57	2.2 × 10^−4^
alr2838	*-*	Unknown protein	−1.18	9.3 × 10^−4^
alr2839	*-*	Glycosyltransferase	−1.17	4.9 × 10^−4^
alr2840	*-*	Glycosyltransferase	−1.38	1.9 × 10^−4^
alr2841	*-*	Unknown protein	−1.32	6.8 × 10^−5^
alr2857	*-*	Unknown protein	−1.22	2.6 × 10^−3^
alr3195	*-*	Probable glutathione S-transferase	−1.04	1.5 × 10^−4^
alr3301	*-*	Unknown protein	−1.05	1.8 × 10^−4^
all3420	*-*	Carboxyl-terminal processing protease	−1.40	3.0 × 10^−3^
alr3698	*hepB*	Heterocyst envelope polysaccharide synthesis protein	−1.35	4.5 × 10^−4^
alr3699	*-*	Similar to glycosyltransferase	−1.00	7.7 × 10^−4^
alr3710	*devB*	Membrane fusion protein of ABC transporter	−1.92	1.9 × 10^−4^
alr3711	*devC*	Substrate-binding protein of ABC transporter	−1.71	9.3 × 10^−6^
all3773	*-*	Serine/threonine kinase	−1.47	5.9 × 10^−3^
all3780	*-*	Similar to kinesin light chain	−1.80	1.4 × 10^−3^
all3793	*-*	Unknown protein	−1.25	3.9 × 10^−3^
all4160	*-*	Probable glycosyltransferase	−1.27	2.3 × 10^−3^
alr4984	*-*	Unknown protein	−1.60	1.4 × 10^−3^
all4991	*desC*	Delta-9 desaturase	−1.06	2.2 × 10^−2^
all5341	*hglT*	Heterocyst-specific glycolipid synthase	−1.65	6.0 × 10^−3^
alr5358	*hetN*	Ketoacyl reductase	−1.34	1.9 × 10^−3^

^a^ Relative ratios of transcript levels in the *sigE* disruptant to those in the wild-type strain at 8 h after nitrogen deprivation are shown in the base 2 logarithm; ^b^ *p* values for the relative ratios were determined by the Student’s *t-*test.

## 4. Concluding Remarks

In the present study, genes regulated by SigC, SigE, and SigG of *Anabaena* PCC 7120 were identified. It was suggested that group 2 sigma factors of *Anabaena* PCC 7120 have at least partially overlapping promoter specificities by genetic analysis of sigma factor genes. In this study, comprehensive analysis of gene expression in mutants of sigma factor genes showed that genes regulated by SigC and SigE partially overlap. It was indicated that *coxA2*, *talB*, *hepB*, all4160, and the HEP island are regulated by both SigC and SigE. The transcript levels of these genes were lower in the *sigC* disruptant than in the WT strain at 8 h after nitrogen deprivation, whereas the expression of these genes was higher in the *sigC* disruptant than in the WT strain after 24 h, suggesting that SigE compensated for the deficiency of SigC. We cannot distinguish between direct and indirect regulation based on the data presented here. Therefore, the possibility that a transcriptional regulator whose expression depends on both SigC and SigE is responsible for the overlap between the genes regulated by SigC and SigE cannot be ruled out, although no gene encoding a transcriptional regulator was downregulated in both the *sigC* and *sigE* disruptants. Genes required for heterocyst differentiation are induced in differentiating cells. SigC and SigE are likely to act cooperatively to fully and rapidly induce these genes in differentiating cells. The expression patterns of *fbp* and *coxB3* were similar to those of genes regulated by both *sigC* and *sigE*. However, *fbp* and *coxB3* were not regulated by SigE, indicating that sigma factors other than SigE can also compensate for the deficiency of SigC. In *Synechocystis* PCC 6803, it is indicated that multiple group 2 sigma factors can recognize the same promoter as the group 1 sigma factor SigA [43]. In *Anabaena* PCC 7120, there are seven group 2 sigma factors. It is proposed that redundancy of transcriptional regulators is not only a selective advantage, but also permits strict regulation of gene expression [44].
